# An asymptomatic postmenopausal uterine perforation with bilateral embedment by a V-shaped intrauterine device: a case report

**DOI:** 10.3389/fmed.2026.1772440

**Published:** 2026-02-05

**Authors:** Xilian Wu, Ruxi Zheng, Tianye Li, Jianwei Zhou, Yaxi Ma

**Affiliations:** 1 Department of Gynecology, The Second Affiliated Hospital, Zhejiang University School of Medicine, Zhejiang University, Hangzhou, China; 2 Department of Obstetrics and Gynecology, Shanghang County Hospital, Longyan, Fujian, China

**Keywords:** fallopian tube, hysteroscopy, intrauterine device, laparoscopy, postmenopause, uterine perforation

## Abstract

**Background:**

Retained intrauterine devices (IUDs) after menopause are common in China, yet their complications may be clinically silent. Uterine atrophy and rigid open-frame designs can permit gradual transmural erosion, leading to seemingly routine removal requests that conceal high-risk extrauterine involvement.

**Case presentation:**

A 54-year-old asymptomatic postmenopausal woman presented for elective IUD removal. Transvaginal ultrasound suggested deep myometrial embedment with suspected serosal extension. During hysteroscopy, the device could be grasped but exhibited extreme traction resistance, raising concern for transmural perforation; the procedure was immediately converted to laparoscopy. Laparoscopy revealed a rare dual-site extrauterine embedment: one arm perforated the posterior uterine wall and was embedded in the pelvic peritoneum adjacent to the left ureter, while the contralateral arm traversed the fundus and was completely impacted within the right tubal isthmus. Right therapeutic salpingectomy and opportunistic contralateral salpingectomy were performed. Under laparoscopic visualization, the IUD was then safely retrieved through the uterine cavity with hysteroscopic guidance. The postoperative course was uneventful.

**Conclusion:**

This case illustrates that postmenopausal IUD retention can culminate in severe, multi-organ-adjacent perforation without symptoms, and that ultrasound may underestimate the extent of extrauterine involvement. In hysteroscopic IUD removal, “extreme traction resistance” should be treated as an intraoperative red-flag prompting immediate cessation and conversion to laparoscopy. A combined hysteroscopic-laparoscopic strategy enables controlled dissection, organ protection, particularly the ureter and tube, and complete retrieval with minimal uterine trauma.

## 1 Introduction

Intrauterine devices (IUDs) are widely used globally due to their high efficacy, long duration of action, reversibility, and affordability. In China, IUDs constitute a vital component of the national family planning policy. According to statistics from the National Health Commission, IUDs account for over 40% of all contraceptive methods used, with cumulative users exceeding 80 million, representing the largest population of IUD users worldwide (1). A pressing public health concern is the large number of IUDs retained in postmenopausal women, particularly open-frame devices (such as V-shaped and T-shaped IUDs) inserted decades ago. It was reported that 40.2% of women had their IUDs removed more than 2 years after menopause (2). Declining estrogen levels after menopause result in uterine atrophy, increasing the risk of embedding, perforation, and displacement (3). Among these, the V-shaped IUD, due to its rigid arms and open-frame design, carries a higher risk of penetrating the uterine wall than closed-loop devices, with potential migration into the abdominal cavity or adjacent organs such as the bladder, even beyond the anterior wall of the bladder (4–8).

Most IUD displacements are asymptomatic and are discovered incidentally during routine examinations or evaluations for unrelated conditions, contributing to delayed diagnosis and management (9, 10). Beyond mechanical complications, increasing evidence suggests that long-term copper IUD use induces chronic endometrial and myometrial alterations, including inflammation, fibrosis, and architectural distortion (11). A recent study demonstrated that copper IUD use significantly reduces the adequacy of endometrial biopsy specimens, particularly in perimenopausal and postmenopausal women, underscoring that prolonged IUD retention may conceal complex intrauterine or transmural pathology even in asymptomatic patients (12). This underscores that prolonged IUD retention may conceal complex pathology even in asymptomatic patients. Although IUD-related complications have been reported, detailed guidance on preoperative evaluation and minimally invasive surgical strategies for asymptomatic cases with severe, multi-site perforation (especially those involving adjacent organs) remains limited.

We present a rare case of asymptomatic bilateral uterine perforation involving both the pelvic peritoneum and fallopian tube caused by a V-shaped IUD in a postmenopausal woman. This report highlights key imaging and intraoperative decision points and proposes a pragmatic, stepwise management strategy relevant to both primary and tertiary care settings.

## 2 Case presentation

A 54-year-old, G1P1, postmenopausal woman presented to the hospital on July 17, 2025, for IUD removal. She was generally healthy and asymptomatic. The IUD had been inserted at a local hospital in 2007, though the specific type was unknown to the patient. Previous routine follow-up ultrasounds had reportedly confirmed normal IUD position.

*Physical examination*: Vital signs were stable. External genitalia were normal; findings were consistent with prior vaginal delivery. Vagina patent; cervix smooth. The uterus was retroverted and atrophic. No palpable masses or tenderness were noted in the adnexa.

**Figure 1:**
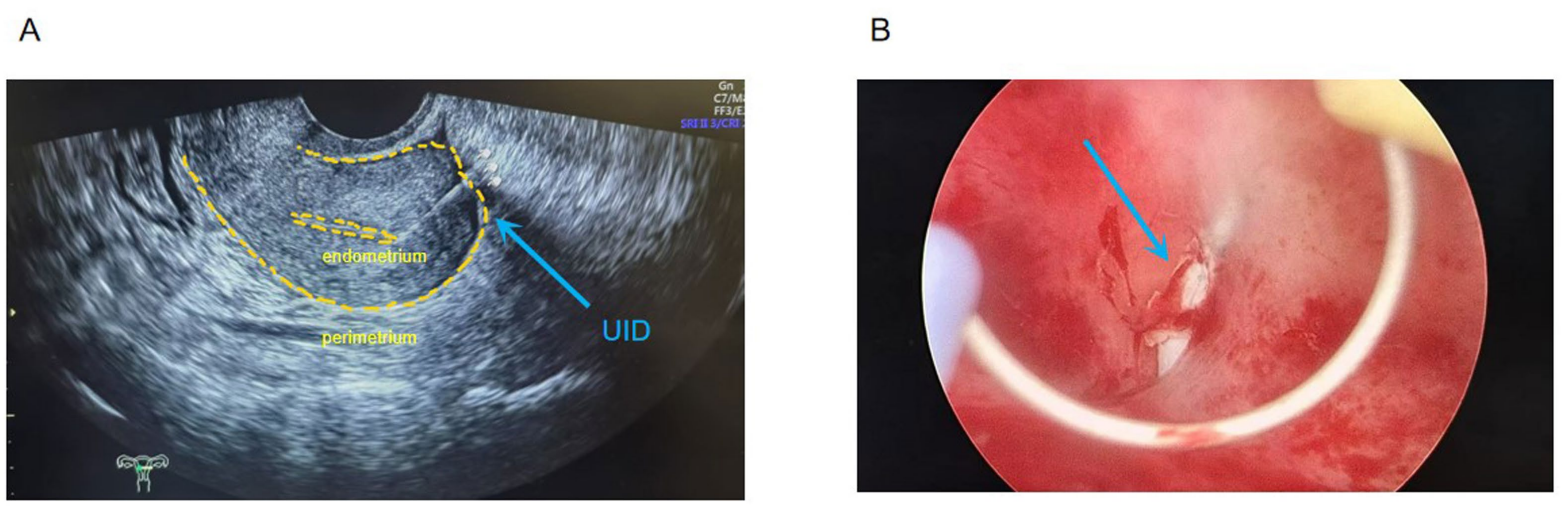
**(A)** Preoperative transvaginal ultrasound showing IUD echogenicity with partial protrusion toward the serosal surface (arrow). **(B)** Hysteroscopic view showing the IUD embedded within the myometrium near the left uterine horn (arrow).

*Preoperative assessment*: Transvaginal ultrasound showed uterine atrophy with echogenicity of an intrauterine device extending into the myometrium and partially protruding beyond the serosal surface (Figure 1A). Routine laboratory tests were unremarkable. Based on the patient’s completely asymptomatic status, the ultrasound impression of apparently localized embedment without clear evidence of adjacent organ involvement, and our institutional day-surgery pathway for postmenopausal IUD removal, additional cross-sectional imaging such as CT or MRI was not initially pursued. This decision reflected a balance between clinical presentation, radiation exposure, cost considerations, and routine practice patterns. However, in retrospect, the absence of preoperative CT limited precise delineation of the extrauterine trajectory of the device and is acknowledged as an important limitation of this case.

After informed consent and preoperative preparation (oral misoprostol for cervical softening), day-surgery removal was planned.

### 2.1 Surgical procedure

**Figure 2:**
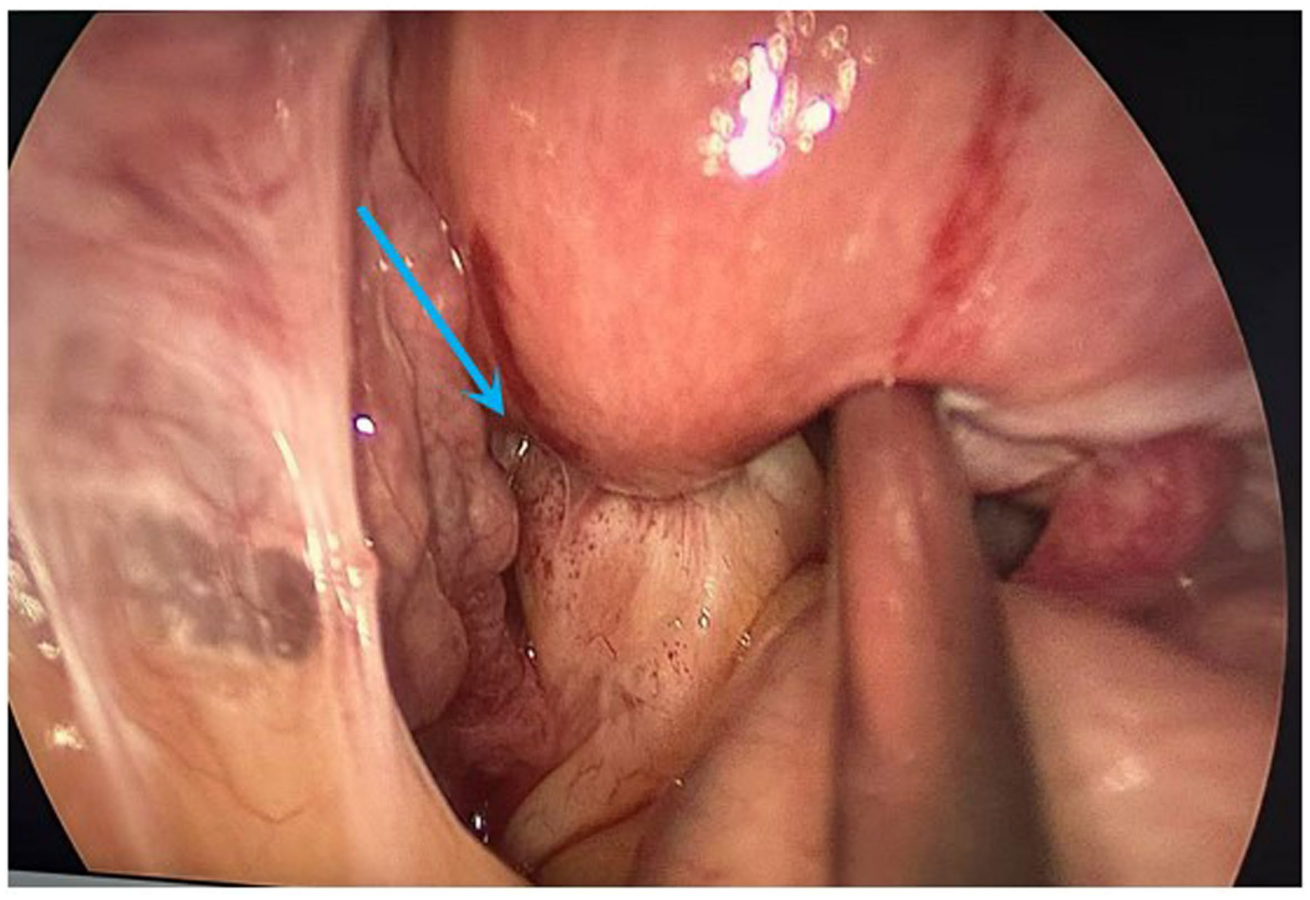
Laparoscopic view demonstrating adhesions between the posterior uterine wall and the pelvic wall (arrow).

**Figure 3:**
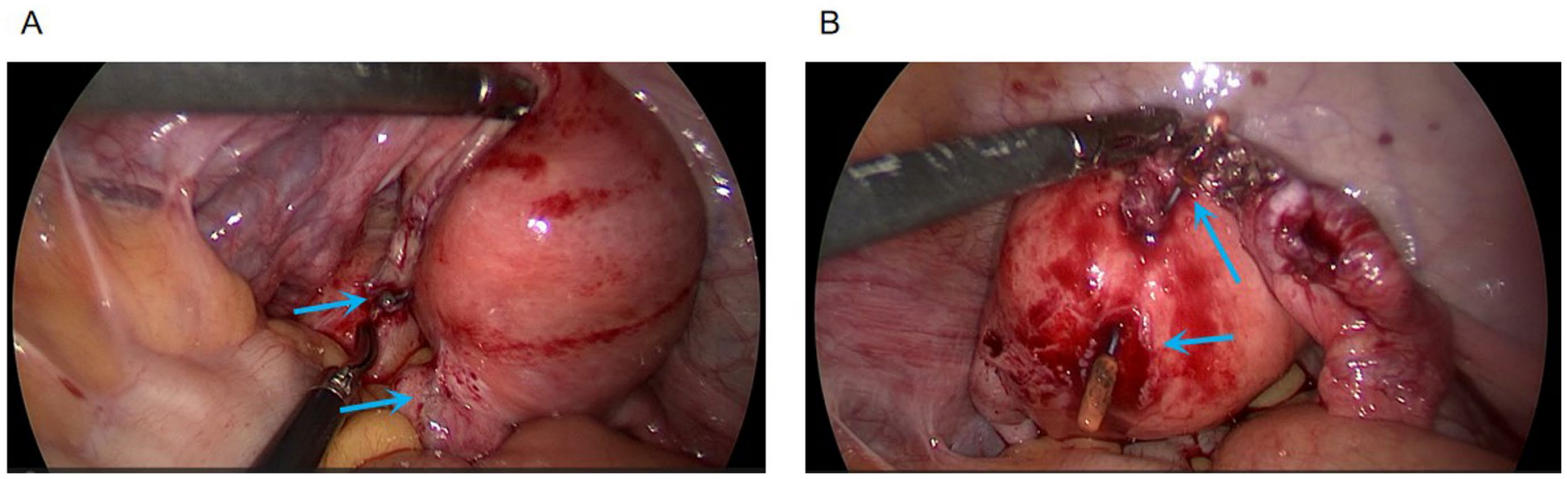
**(A)** Perforation site of the V-shaped IUD at the posterior uterine wall (arrow). **(B)** IUD arm traversing the fundus and completely impacted within the right tubal isthmus (arrow).

**Figure 4:**
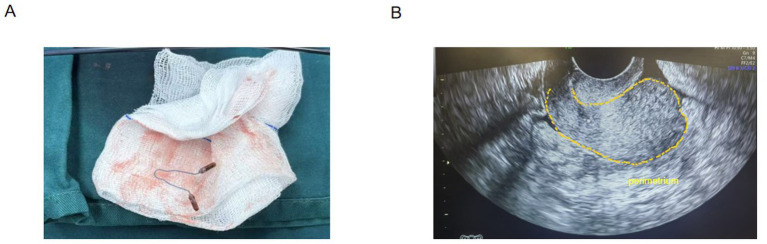
**(A)** Intact V-shaped IUD after removal *via* the uterine cavity. **(B)** Follow-up color Doppler ultrasound obtained 1 month postoperatively showing no abnormalities.

1) *Hysteroscopic exploration*: Uterine cavity depth was 7 cm. Hysteroscopy revealed a normal cavity configuration with atrophic endometrium. An IUD was seen completely embedded within the myometrium near the left uterine horn at the fundus (Figure 1B). When the device was grasped with forceps, extreme traction resistance was encountered. Penetrating embedment with possible extrauterine involvement (e.g., bowel, bladder, fallopian tube, or adjacent vessels) was suspected. To avoid organ injury and hemorrhage, the procedure was terminated and converted to laparoscopy.
2) *Laparoscopic exploration*: Adhesions were noted between the posterior uterine wall adjacent to the left uterine horn and the pelvic wall, and between the right uterine horn and the right tubal isthmus (Figure 2). After careful adhesiolysis, one arm of the V-shaped IUD was found to have perforated the posterior uterine wall, with the distal end embedded in pelvic peritoneum near the left ureter and the uterosacral ligament; the left ureter was intact. The contralateral arm had traversed the uterine fundus and was fully impacted within the right tubal isthmus (Figures 3A,B).
3) *Definitive surgery and device retrieval*: Because the IUD caused perforating injury to the right fallopian tube, right salpingectomy was performed. After discussion with the patient’s family, opportunistic contralateral salpingectomy was performed. Given the thickened arms of the V-shaped IUD and the main body remaining intrauterine, abdominal extraction posed higher risk of uterine trauma and would have required a uterine incision. Under laparoscopic visualization, the hysteroscope was reintroduced; the intrauterine portion of the device was gently grasped with foreign-body forceps and removed via the uterine cavity, achieving complete retrieval of the intact V-shaped IUD (Figure 4A).
4) *Postoperative course*: Prophylactic antimicrobial therapy was administered. The patient tolerated clear liquids 6 h postoperatively. Recovery was uneventful; she was discharged the next day. Ultrasound at 1-month follow-up showed no abnormalities (Figure 4B). She remained asymptomatic.

## 3 Discussion

This case is notable for three uncommon and clinically instructive features. First, it demonstrates that severe uterine perforation and extrauterine embedment may remain entirely asymptomatic, challenging the assumption that clinically silent IUDs are benign. Secondly, it reveals a dual-site extrauterine involvement characterized by two distinct trajectories: posterior wall perforation into the ureter-adjacent pelvic peritoneum and transfundal extension leading to complete impaction within the tubal isthmus. This complex anatomical pattern is seldom fully appreciated prior to surgery. Third, it underscores a critical intraoperative decision point: extreme traction resistance during hysteroscopic removal should be regarded as a red flag for transmural fixation, where persistence may convert a controlled procedure into iatrogenic injury.

### 3.1 Risk factors for IUD displacement and perforation

Risk factors can be broadly categorized as follows. Patient-related factors include low-estrogen states such as lactation, postpartum status, and postmenopause, which cause thinning and atrophy of the uterine wall and increase perforation risk (3, 13). Uterine retroversion, fibroids, and congenital anomalies may further increase insertion difficulty and focal pressure (14, 15). Device-related factors include open-frame designs (such as V-shaped or T-shaped IUDs), which have greater perforation potential due to rigid arms compared with closed-loop devices (5, 16). Prolonged retention is also an important risk factor for the IUD embedment (17). Operator-related factors include insertion by inexperienced practitioners, which may result in immediate or unrecognized perforation (14, 18). Other contributing factors include prior intrauterine procedures or cesarean section, which may weaken the myometrium or create adhesions (1, 8, 19). In this patient, postmenopausal atrophy, long-term retention of an open-frame V-shaped IUD, and uterine retroversion likely acted synergistically to produce severe bilateral perforation.

### 3.2 Stepwise minimally invasive strategy for complex embedment

Hysteroscopy is generally considered the first-line approach for removal of embedded IUDs (1). However, intraoperative “extreme traction resistance” represents a practical and critical warning sign of penetrating or transmural embedment and should prompt immediate cessation to avoid device fracture, uterine rupture, or injury to adjacent organs. When such resistance is encountered, timely conversion to laparoscopy enhances procedural safety by allowing full pelvic inspection, adhesiolysis, assessment of adjacent organs, and controlled retrieval (9). In cases where the device straddles the uterine wall, a combined hysteroscopic-laparoscopic approach enables coordinated internal and external manipulation, facilitating safe and complete removal while minimizing uterine trauma.

### 3.3 Managing organ injury and tailoring care for postmenopausal patients

Management of perforated or migrated IUDs should follow individualized, multidisciplinary principles aimed at complete foreign-body removal and appropriate repair, in accordance with guideline recommendations (20, 21). Surgical strategies depend on the organs involved and may require collaboration with urology or general surgery for bladder or bowel injury (22–24). Meticulous imaging assessment and surgical planning are essential. In the present case, the proximity of one IUD arm to the ureter and complete impaction within the tubal isthmus highlight the importance of careful dissection and intraoperative visualization. For postmenopausal women without fertility desire, therapeutic salpingectomy for the injured tube and opportunistic contralateral salpingectomy may be considered, aligning with ovarian cancer risk-reduction strategies (25, 26). Moreover, emerging evidence indicates that long-term copper IUD use induces chronic endometrial changes and reduces the adequacy of endometrial sampling, reinforcing that apparently routine IUD removals in postmenopausal women may conceal complex intrauterine or transmural pathology (12).

### 3.4 Preoperative imaging: ultrasound versus CT

Transvaginal ultrasound is the preferred initial imaging modality for assessing IUD position because of its accessibility, low cost, and lack of radiation exposure. However, ultrasound may underestimate serosal penetration and frequently fails to delineate extrauterine extension or involvement of adjacent organs. In this case, ultrasound suggested partial serosal protrusion but did not predict tubal impaction or ureter-adjacent involvement.

For high-risk patients, such as postmenopausal women, those with long-standing open-frame IUDs, or ultrasound findings suggestive of deep embedment or serosal extension, contrast-enhanced pelvic CT with three-dimensional reconstruction provides a valuable spatial “roadmap.” CT can clearly define the relationship between the IUD, uterine wall, fallopian tubes, ureters, bowel, and major vessels, thereby facilitating operative planning and anticipation of a combined hysteroscopic-laparoscopic approach (9, 27). In addition, complex cases may benefit from multidisciplinary discussion involving gynecologists, radiologists, and, when appropriate, urologists or general surgeons, to optimize interpretation of imaging findings and enhance patient safety.

### 3.5 Public health context

Given China’s large population of long-term IUD users, a substantial number of postmenopausal women will require IUD removal in the coming years. Asymptomatic status, limited awareness, or fear of procedures often delay removal beyond 1 year after menopause, allowing chronic pressure from an atrophic uterus to erode the myometrium and cause silent perforation. Strengthening education and routine screening in primary care, particularly in rural areas, is critical to reducing the incidence of severe complications.

### 3.6 Considerations for low-resource settings

While the combined hysteroscopic-laparoscopic approach described requires resources not universally available, key management principles remain transferable to low-resource settings. Nevertheless, several pragmatic principles are broadly applicable. Timely removal of IUDs after menopause should be emphasized through patient education. At minimum, pelvic ultrasound should be performed before attempted removal. If imaging suggests malposition or deep embedment and advanced imaging or laparoscopy is unavailable, forceful blind traction should be avoided, and referral to a higher-level center should be considered. In facilities with hysteroscopy but without laparoscopy, extreme traction resistance should be treated as a stop signal rather than an indication to increase force. These principles underscore that while the exact operative strategy may not be universally feasible, the underlying decision-making framework remains widely applicable.

### 3.7 Limitations

This report has several limitations. First, as a single-case report, its findings have limited generalizability. Second, the decision to forgo preoperative cross-sectional imaging (CT or MRI) was based on the initial clinical and ultrasonographic assessment; however, this restricted the precise preoperative delineation of the complex extrauterine trajectory of the IUD, which was more extensive than anticipated. This experience underscores that even in asymptomatic patients with ultrasound findings suggestive of serosal penetration, advanced imaging can provide critical spatial information. Third, the follow-up duration was relatively short; longer-term surveillance would be valuable to confirm the absence of delayed complications. Finally, the successful combined hysteroscopic-laparoscopic approach described herein requires specialized endoscopic equipment and surgical expertise, which may limit its immediate applicability in low-resource settings where such resources are scarce.

## 4 Conclusion

Postmenopausal IUD retention should not be regarded as a benign finding. As illustrated by this rare case of asymptomatic dual-site extrauterine embedment of a V-shaped IUD, serious perforation may progress silently and evade accurate preoperative assessment by ultrasound alone. For high-risk patients with suspected serosal extension, preoperative CT with 3D reconstruction can improve procedural planning. Intraoperatively, extreme traction resistance during hysteroscopic extraction should be treated as a mandatory stop signal and should prompt timely conversion to laparoscopy. A combined hysteroscopic-laparoscopic approach enables safe dissection, minimizes uterine trauma, and ensures complete retrieval when adjacent organ involvement is possible.

## Patient perspective (optional)

“I had no discomfort and was surprised to learn the IUD had perforated the uterus and tube. I'm grateful it was removed with minimally invasive surgery. I now understand the importance of timely IUD removal after menopause.”

## Data Availability

The original contributions presented in the study are included in the article/Supplementary material, further inquiries can be directed to the corresponding authors.
